# Multiwalled carbon nanotubes-jasmonate interactions enhance biomass production by regulating reactive oxygen species homeostasis, δ-aminolevulinic acid/glutamate-1-semialdehyde metabolism, source–sink balance, and defense responses under chromium stress in soybean

**DOI:** 10.3389/fpls.2026.1806073

**Published:** 2026-05-08

**Authors:** Gulsah Bengisu, Umran Atay, Buse Tagay Bice, Abdulrahman A. Alatar, Mohammad Faizan, Muhammad Faheem Adil, Mohammad Faisal

**Affiliations:** 1 Department of Field Crops, Faculty of Agriculture, Harran University, Sanliurfa, Türkiye; 2 Department of Machinery and Metal Technologies Machinery Program, Vocational School of Organized Industrial Site, Mardin Artuklu University, Mardin, Türkiye; 3 Çatalpınar District Directorate of Agriculture and Forestry, Ordu, Türkiye; 4 Department of Botany & Microbiology, College of Science, King Saud University, Riyadh, Saudi Arabia; 5 Botany Section, School of Sciences, Maulana Azad National Urdu University, Hyderabad, India; 6 College of Agriculture and Biotechnology, Zhejiang University, Hangzhou, China

**Keywords:** nanotubes, nutrient imbalance, photosynthetic pigments, phytohormone, seed priming, stress tolerance

## Abstract

Chromium (Cr) contamination severely reduces soybean growth by impairing photosynthesis, nutrient uptake, and osmolyte balance, posing a significant threat to food security. This study evaluated the synergistic effects of multiwalled carbon nanotubes (CNTs) and jasmonic acid (JA) in mitigating chromium (Cr) stress in soybean plants. Chromium stress markedly reduced delta amino levulinic acid (64.33%), Glutamate-1-semialdehyde (66.87%), carotenoids (58.11%), RuBisCo activity (73.01%), and total soluble sugar (48.34%), while increasing oxidative damage as indicated by higher malondialdehyde and electrolyte leakage. Co-application of CNTs and JA effectively mitigated Cr-induced oxidative stress in soybean by reducing hydrogen peroxide and malondialdehyde levels. It also improved SPAD chlorophyll, PSII efficiency, net photosynthetic rate, and transpiration rate, along with increasing flavonoid, protein, and iron content, while lowering Cr accumulation. Chromium stress significantly reduced total phenols (51.11%), flavonoids (57.12%), free amino acids (50.06%), carbon (46.12% & 53.07%), phosphorus (53.34% & 50.22%), copper (39.64% & 41.28%), and zinc (52.03% & 63.74%). However, CNTs and JA application reversed these effects by enhancing total phenols, flavonoids, free amino acids, carbon, phosphorus, copper, and zinc. Additionally, Cr accumulation in both shoots and roots was significantly reduced with CNTs and JA supplementation. Therefore, CNTs and JA mitigated Cr toxicity by improving growth, photosynthesis, antioxidant enzymes activity, and nutritional homeostasis. These findings demonstrate the potential of CNTs and JA as sustainable nano-hormonal based strategy for reducing Cr accumulation in soybean and safeguarding food security.

## 1 Introduction

In recent decades, rapid industrialization and increasing human activities have led to a worrying rise in the release of toxic metals into agricultural soils. This is now a global problem (Wang et al., 2019). These heavy metals do not break down and will stay in the environment forever (Sirhindi et al., 2015). The growing problem of these metals building up in the parts of plants that people and animals eat is a direct threat to their health through the food chain (Zhao et al., 2019). Among various metals, chromium (Cr) is one of the non-essential carcinogenic pollutants among toxic metals, and it is well known for having dangerous effects on both living things and the environment (Kumar et al., 2019; Saud et al., 2022; Singh et al., 2017). Chromium is well known for having dangerous effects on both living organisms and the environment. In living beings, especially in its hexavalent form (Cr^6+^), Cr is highly toxic and can cause severe health problems such as oxidative stress, DNA damage, respiratory disorders, skin irritation, kidney and liver damage, and even carcinogenic effects in humans and animals (Chakraborty et al., 2022). In plants, excessive chromium accumulation inhibits seed germination, reduces photosynthesis, disrupts nutrient uptake, damages cellular structures, and ultimately suppresses plant growth and productivity. Environmentally, chromium contamination in soil and water alters microbial communities, decreases soil fertility, contaminates groundwater, and enters the food chain through bioaccumulation, posing long-term ecological and health risks (Qureshi et al., 2024; Yonghui et al., 2025). Chromium is a major industrial pollutant that is released into the environment in large amounts because it is used a lot in tanning, smelting, metal plating, refractory materials, battery production, pesticides, and fertilizers (Singh and Prasad, 2019). Cr(III) and Cr(VI) are the most stable and common valences of Cr. The latter is known to be very carcinogenic and mutagenic for people and animals (Farid et al., 2018). Cr in soil and water messes up the balance of water and minerals in plants and makes crops much less productive (Ahmad et al., 2020). Additionally, elevated Cr accumulation induces irreversible physiological, biochemical, anatomical, and ultrastructural modifications in plants (Ahmad et al., 2020). Chromium toxicity inhibits seed germination and early seedling establishment mainly by inducing oxidative stress and disrupting metabolic activities. Excess Cr increases the production of reactive oxygen species (ROS), which damage cellular membranes, proteins, and nucleic acids, thereby impairing enzymatic processes required for germination (Singh et al., 2020). It also disturbs hormonal balance and inhibits hydrolytic enzymes responsible for mobilizing seed reserves, leading to delayed germination. Furthermore, Cr interferes with cell division and elongation, restricting root and shoot development and reducing nutrient and water uptake. Chromium also impairs chlorophyll biosynthesis and damages the photosynthetic electron transport system, resulting in chlorosis and reduced photosynthetic efficiency. Additionally, Cr disrupts transpiration and respiration and inactivates key metabolic enzymes, ultimately suppressing overall plant growth and development (Jiahui et al., 2025; Zhao et al., 2019).

Nanotechnology is an emerging tool for sustainable agriculture, which is expected to transform conventional farming into precision farming. Precision farming is a balanced way to boost crop yields by keeping an eye on environmental factors and taking carefully planned steps based on each one (Chen and Yada, 2011; Singh et al., 2025; Sultan et al., 2026). Nanotechnology could help crops grow better while keeping the environment stable, ecologically balanced, and economically stable (Acharya and Pal, 2020; Bilge et al., 2025). Several researchers have recently concentrated on nanotechnology-based agriculture to enhance agricultural productivity by facilitating efficient nutrient delivery, regulating nutrient levels, minimizing mobile nutrient losses, creating slow-release fertilizers, and augmenting the accessibility of scarce nutrients (Lowry et al., 2019; Elhakem et al., 2025). Nanomaterials are thought to be the best way to start the agri-nanotech revolution because they are so small (less than 100 nm) that they can get through biological barriers and into plant tissues through foliar or root application. This means that they can deliver nutrients and pesticides in new and more effective ways (Shah and Aslam, 2025; Poddar et al., 2018; Faisal et al., 2025).

CNTs have emerged as a promising nanotechnology in modern agriculture, offering innovative solutions to enhance crop productivity, stress tolerance, and sustainability (Faizan et al., 2021). They can interact with plant systems at many levels because of their unique physicochemical properties, like a high surface area, chemical inertness, and the ability to cross biological membranes. Recent research has shown that CNTs can have a big effect on important physiological and biochemical processes, such as germination of seeds, taking in nutrients and water, changing gene expression, and delivering agrochemicals or genetic material to specific areas. CNTs, which include single-walled carbon nanotubes (SWCNTs) and multi-walled carbon nanotubes (MWCNTs), are one of the most common types of nanomaterials made around the world (Zhai et al., 2015). CNTs are used a lot in wastewater treatment, biomedicine, and environmental protection. They also have great potential in agriculture because of their unique properties, such as their ability to absorb large amounts of light and infrared light (Ali et al., 2021). Studies have shown that MWCNTs can raise the levels of calcium and iron in maize seedlings (Tiwari et al., 2013), encourage nodulation and nitrogen fixation in the legume Lotus japonicus (Yuan et al., 2017), boost the growth and yield of wheat and rice (Joshi et al., 2020), and even help maize seedlings grow by coordinating carbon and nitrogen metabolism (Hu et al., 2021).

CNT priming has become a promising method for improving seed germination and early plant growth. CNTs interact with the seed coat to make nano-channels that help the seed take in more water and nutrients, which is important for the early stages of germination (Mousavi et al., 2025). Compared to untreated controls, these interactions greatly improve imbibition, shorten the average time it takes for seeds to germinate, and boost seedling vigor. Pathak et al. (2023) found that MWCNTs made tomato (*Solanum lycopersicum*) and barley (*Hordeum vulgare*) seeds germinate much better, with a 20–30% increase in the percentage of seeds that germinated. Yadav et al. (2024) found that exposing wheat (*Triticum aestivum*) to CNT increased the rate at which it absorbed water, which led to faster radicle emergence and better seedling establishment. In maize (Zea mays), CNT priming decreased the mean germination time by around 18% compared to control groups (Mousavi et al., 2025), a significant enhancement, especially under suboptimal environmental conditions with limited water availability. SWCNTs have exhibited species-specific effects on germination, with concentrations between 10 and 40 mg/L markedly enhancing germination rates in tall fescue, pepper, and salvia.

In recent years, significant endeavors have been undertaken to concoct diverse methodologies aimed at enhancing plant resilience to heavy metal toxicity (Adrees et al., 2015; Mostofa et al., 2015). One of these is the use of phytohormones from outside the plant to activate its defense systems against Cr stress. This could be a useful and cheap way to do it. Jasmonic acid (JA) is a common signaling molecule that has many effects on plant growth and development, including controlling plant defense systems and interacting with other phytohormones (Li et al., 2017; Mir et al., 2018). Changes in endogenous levels of JA in plants have been identified as an adaptive strategy for protection against abiotic stresses (Najafi Kakavand et al., 2019). Foliar application of JA mitigated salinity and drought stress, enhancing plant growth by modulating oxidative stress, increasing the synthesis of osmo-regulators, and facilitating the activity of ROS scavenging enzymes (Farhangi-abriz and Ghassemi-golezani, 2018). Some studies have indicated that low concentrations of jasmonates mitigate the impact of abiotic stresses (Mir et al., 2018), whereas high concentrations impede growth and photosynthesis and expedite senescence (Yan et al., 2015).

Metal stress leads to the production of reactive oxygen species (ROS), which significantly compromises cell membrane integrity by hastening lipid peroxidation (Gao et al., 2018). Oxidative stress diminishes the functionality of photosystem I (PSI) and photosystem II (PSII) by impairing the electron transport chain and compromising chloroplast integrity (Zaid et al., 2019). To protect themselves from the harmful effects of ROS, plants have a complex antioxidant defense system that gets rid of the extra ROS (Gill and Tuteja, 2010). The antioxidant defense system consists of enzymatic antioxidants (catalase, CAT; ascorbate peroxidase, APX; superoxide dismutase, SOD; glutathione reductase, GR; glutathione S-transferase, GST; glutathione peroxidase, GPX; monodehydroascorbate reductase, MDHAR; and dehydroascorbate reductase, DHAR) and non-enzymatic antioxidants (glutathione, GSH; ascorbate, AsA; and α-tocopherol) (Gill and Tuteja, 2010). However, the production of ROS frequently exceeds the antioxidant capacity of plants under extreme conditions, resulting in oxidative damage. Most crop plants’ ability to handle stress is linked to how well their oxidative system works (Najafi Kakavand et al., 2019). Consequently, enhancing the efficacy of antioxidant defense mechanisms in plants is essential for promoting their adaptive capacity under stress conditions (Kamran et al., 2020). Prior research has indicated the generation of cytotoxic methylglyoxal (MG) in response to metal stress (Zaid et al., 2019). The toxicity of MG is well-documented to exacerbate ultra-structural damage, oxidative injury to cellular components, mutations, and the inactivation of proteins and DNA (Jianxin et al., 2025; Kamran et al., 2020). Plants counteract MG toxicity through the glyoxalase (Gly) defense system, which consists of two enzymes: glyoxalase I (Gly I) and glyoxalase II (Gly II), which convert MG into D-lactate (Alam et al., 2014). It is known that plants are more resistant to different types of abiotic stress when their Gly and antioxidant defense systems are well-regulated and work together (Mir et al., 2018).

The primary objective of this study was to investigate the interactive effects of CNTs and JA on soybean plants exposed to chromium stress, with particular emphasis on their role in regulating reactive oxygen species homeostasis, modulating source–sink metabolism, enhancing nutrient assimilation, and strengthening antioxidant and defense responses to improve overall stress tolerance.

## 2 Material and methods

### 2.1 Growth conditions and experimental layout

Soybean seeds were surface-sterilized with sodium hypochlorite for 5 min and then washed with double-distilled water (DDW). The sterilized seeds were sown in pots, which were filled with soil and manure, and then allowed to grow under natural environmental conditions. The basic physicochemical properties of the soil were as follows: total selenium (Se), 0.25 mg kg^-1^, cadmium (Cd), 0.318 mg kg^-1^, total phosphorus (P), 0.92 g kg^-1^, total nitrogen (N), 2.16 g kg^-1^, total potassium (K), 3.1 g kg^-1^, organic matter, 35.2 g kg^-1^, and pH, 7.1. Round plastic pots were used, each filled with 8 kg of soil. Chromium (Cr) stress (150 µM), MWCNTs (100 ppm), and jasmonate (1 µM) were applied at the seed stage by soaking the seeds in their respective solutions for 8 h prior to sowing. Following transplantation, soybean plants were irrigated daily according to their respective treatment regimes. Each pot contained four healthy soybean plants, and each treatment was replicated five times (n = 5) using a completely randomized design (CRD). For this experiment, a total of 35 pots were needed. These 35 pots were divided in 7 sets, with 5 pots in each set. Seven treatments were maintained as follows:

T1: Control.
T2: Cr (150 µM).
T3: CNTs (100 PPM).
T4: JA (1 µM).
T5: Cr (150 µM) + CNTs (100 PPM).
T6: Cr (150 µM) + JA (1 µM).
T7: Cr (150 µM) + CNTs (100 PPM) + JA (1 µM).

At 40 days after sowing (DAS), corresponding to the vegetative growth stage, plants were harvested for the evaluation of growth, physiological, and biochemical parameters.

### 2.2 Characterization of multi walled CNTs

Multi walled CNTs were morphologically and structurally characterized using scanning electron microscopy (SEM). SEM (TM-1000, Hitachi, Japan) analyses were performed to evaluate particle size and surface morphology. SEM images demonstrated a homogeneous distribution of the CNTs. For SEM analysis, CNTs were fixed onto an aluminum stub using carbon double-sided adhesive tabs and coated with a thin conductive palladium layer. 

### 2.3 Plant height, fresh and dry weight of plant

Plant height was measured by using a scale. Fresh weight of whole plant was taken immediately and entire water was blot dried before recording the weight. Plants were dried in oven for 72 h at 60 degree Celsius (°C) to measure the dry weight. Once the samples had reached constant mass, they were weighed using an electronic analytical balance (EK-120, A&D Co., Ltd., Tokyo, Japan).

### 2.4 Estimation of photosynthetic pigments and their precursors

#### 2.4.1 Glutamate 1-semialdehyde

Method described by Kannangara and Schouboe (1985) was followed for estimating the GSA content. Fresh 200 mg leaf tissue was homogenized in 0.1 N HCl however another set from each treatment was incubated in 500 µM gabaculine under light for 4 h and followed by extraction in 0.1 N HCl. Homogenate was centrifuged at 15 000g followed by addition of HCl and 3-methyl-2-benzothiazolinone hydrazone (MBTH) to the supernatant. After incubating the mixture for 2 min in boiling water bath, the samples were cooled and FeCl_3_ was added. Optical density was taken at 620 nm.

#### 2.4.2 δ-amino levulinic acid

For determination of δ-ALA content, two separate sets from each treatment were taken and one set was incubated in levulinic acid (60 mM) under light for 4 h while as other set was extracted immediately in sodium acetate buffer (1 M, pH 4.6). After centrifuging the extract at 15000g, supernatant mixed with acetyl-acetone and boiled. After cooling Ehrlich’s reagent, glacial acetic acid and perchloric acid were added and samples were thoroughly mixed. After incubating the samples for 10 min, optical density was taken at 555 nm.

#### 2.4.3 SPAD chlorophylls

The chlorophyll content of SPAD was measured between 10:00 AM and 11:00 AM using a Minolta chlorophyll meter (SPAD-502; Konica Minolta Sensing Inc., Tokyo, Japan).

#### 2.4.4 Carotenoids

For estimation of carotenoids fresh leaf tissue was extracted in acetone and absorbance recorded at 480, 645 and 663 nm.

### 2.5 Determination of photosynthetic performance

#### 2.5.1 Gas exchange parameters

Photosynthetic gas exchange parameters including net photosynthetic rate (P_N_), intercellular CO_2_ concentration (Ci) and stomatal conductance (gs) were measured using portable photosynthetic apparatus Li-6400 (LI-COR Inc., USA). During the measurements, the environmental conditions inside the leaf chamber were maintained constant, with an ambient temperature of 25± 2 °C, relative humidity of 85 ± 5%, CO_2_ concentration of 600 μmol mol^−1^, and a photosynthetic photon flux density (PPFD) of 800 μmol m^−2^ s^−1^ provided by a red/blue LED light source.

#### 2.5.2 PSII activity

For measuring Fv/Fm, modulated chlorophyll fluorometer (PAM 2500; Walz, Germany) was used and leaves were dark adapted for 25 min.

#### 2.5.3 Rubisco activity

For assaying activity of Rubisco, method described by Sharwood et al. (2016) was followed. Briefly, fresh 500 mg leaf tissue was ground in chilled extraction buffers which contained 50 mM Tris-HCl (pH 8.0), 10 mM β-mercaptoethanol, glycerol (12.5%), 1 mM EDTA, 10 mM MgCl_2_ and 1% polyvinylpyrrolidone (PVP). Homogenate was centrifuged at 15,000g for 10 min and supernatant was collected and used for measuring the activity of Rubisco. Assay mixture contained 10 mM Tris-HCl (pH 8.0), 10 mM MgCl_2_, 1 mM EDTA, 0.2 mM NADH, 20 mM NaHCO_3_, 5 mM dithiothreitol, 5 mM ATP, 10 U ml^−1^ of each glyceraldehyde- 3-phosphodehydrogenase and 3-phosphoglycerate kinase, and 10 mM ribulose 1, 5-bisphosphate (RUBP). Optical density was taken at 340 nm and protein content was estimated according to Lowry et al. (1951).

### 2.6 Oxidative stress markers

For determination of hydrogen peroxide (H_2_O_2_), fresh leaf tissue was homogenized in trichloro acetic acid (TCA) and supernatant was mixed with potassium phosphate buffer (pH 7.0) and potassium iodide. Absorbance was measured at 390 nm (Velikova et al., 2000). For measuring the lipid peroxidation content of malondialdehyde (MDA) formed after reacting the supernatant with thiobarbituric acid was measured at 532 and 600 nm (Heath and Packer, 1968). Activity of lipoxygenase (LOX; EC 1.13.11.12) was assayed according to Doderer et al. (1992) using linoleic acid as substrate. Absorbance was taken at 234 nm.

### 2.7 Antioxidant enzymes

0.1 g of leaf tissue was crushed in 50 mM phosphate buffer (pH 7.0) containing 0.1 mM ethylene diamine tetra acetic acid (EDTA), 0.2% Triton X-100–1 mM phenyl methane sulfonyl fluoride (PMSF), and 2% PVPP (polyvinylpolypyrrolidone) in a pre-chilled mortar and pestle, centrifuged the mixture for 20 min at 13,000 ×g at 4 °C. The supernatant was collected for antioxidant enzyme assays.

#### 2.7.1 Superoxide dismutase

Superoxide dismutase (SOD; EC 1.15.1.1) was assayed by Dhindsa et al. (1981) in a 3 mL reaction mixture containing 0.1 mL enzyme extract, and an equal volume of 1 M NaCO_3_ in a 50 mM phosphate buffer, 13 mM methionine, Nitro Blue Tetrazolium Chloride (75 mM), EDTA (0.1 mM), and riboflavin (2 mM). The tubes were placed under 30 W florescent lights for 10 min. The activity was measured at 560 nm. It is the ability of the enzyme to inhibit the photochemical reduction of NBT. The results were expressed as EU mg^−1^protein.

#### 2.7.2 Catalase

Catalase (CAT; EC 1.11.1.6) was assayed by Aebi (1984). The decomposition of H_2_O_2_ at 240 nm (E = 39.4 mM cm^−1^) in 1 mL reaction mixture containing 50 mM phosphate buffer (pH 7.0), enzyme, and 10 mM H_2_O_2_. It was calculated using the extinction coefficient of 0.036 mM^−1^ cm^−1^, and its activity was expressed as EU mg^−1^ protein.

#### 2.7.3 Ascorbate peroxidase

Ascorbate peroxidase (APX; EC 1.11.1.11) activity was assayed by the method of Nakano and Asada (1981). The extraction was done using potassium phosphate buffer (50 mM). The reaction mixture (1 mL) contained 0.1 mM EDTA, 0.5 mM ascorbate, and enzyme extract (50 µL). The reaction was initiated by addition of 10 µl of 10% (v/v) H_2_O_2_, and the oxidation rate of ascorbic acid was estimated by following the decrease in absorbance at 290 nm for 3 min on a UV–Vis spectrophotometer. An extinction coefficient of 2.8 mM^−1^ cm^−1^ was used for the calculation of APX activity. The results were expressed as EU mg^−1^ protein.

### 2.8 Electrolyte leakage estimation

The level of electrolyte leakage (EL) was determined to assess the damage to leaf membranes. This involved immersing small leaf fragments in deionized water. The initial electrical conductivity (EC) reading was recorded after incubating the sample for two hours at 32 °C, followed by a subsequent reading after further incubation at 121 °C for 20 min (Dionisio-Sese and Tobita, 1998).

### 2.9 Estimation of cellular metabolites

#### 2.9.1 Estimation of phenols

Method of Singleton and Rossi (1965) was followed for estimation of total phenols. Briefly, extraction of dry powdered tissue was carried in methanol and after centrifugation the supernatant was mixed with Folin–Ciocalteu reagent. Optical density was taken at 765 nm and gallic acid was used for calculation.

#### 2.9.2 Total flavonoid

The method of Zhishen et al. (1999) was followed for flavonoid content estimation. 100 µL of the extract was prepared in methanol. From this extract, 1 mL of the sample was taken and 4 mL of DDW was added for dilution, followed by the addition of 0.3 mL of 5% NaNO_3_. After 6 min, 0.3 mL of 10% AlCl_3_ was added and allowed to stand for 10 min. To this mixture, 2 mL of NaOH solution (1 mM) was added. A final volume of 5 mL was made by diluting it with DDW, and test tube was left for color development for 15 min. Absorbance was read at 510 nm. The calibration curve was used to calculate the total flavonoid content, and the results were expressed as µg mg^-1^ Rutin equivalent.

#### 2.9.3 Total free amino acid

Total free amino acid content in soybean leaves was determined using the ninhydrin-based colorimetric assay following Yemm and Cocking (1955). Fresh leaf tissue was homogenized in 80% (v/v) ethanol and centrifuged to extract free amino acids. The collected supernatants were pooled and evaporated to remove ethanol, yielding a concentrated extract. An aliquot of this extract was reacted with ninhydrin reagent in the presence of a suitable buffer, commonly sodium citrate (pH ≈ 5.0), and heated in a boiling water bath for 15–20 min. After cooling to room temperature, the absorbance of the developed purple–blue chromophore was recorded at 570 nm using a spectrophotometer. Total free amino acid concentration was calculated from a standard calibration curve prepared with a known amino acid (e.g., leucine or glycine) and expressed as mg g^-^¹ fresh weight.

#### 2.9.4 Total soluble protein

The total protein content was done by following the method of Bradford (1976). 0.5 g of leaf material was taken and homogenized in 1 mL of phosphate buffer. The mixture was centrifuged at 8000 rpm for 10 min. 0.5 mL of supernatant was added to an equal amount of pre-chilled 20% TCA and again centrifuged at 8000 rpm for 10 min. The supernatant was discarded, the pellet was washed with acetone, and then it was dissolved in 1 mL of 0.1 N NaOH. 5 mL of Bradford reagent was added into 1 mL of aliquot and mixed properly. Tubes were covered and kept in the dark for 10 min. Absorbance was then taken at 595 nm.

#### 2.9.5 Total soluble sugar

Total soluble sugar estimation was done following the method of Dey (1990), in which 0.1 g of fresh leaf sample was taken and then 10 mL of 90% ethanol was added. The mixture was incubated at 60 °C for 1 h, and then 1 mL of 5% phenol was added to 1 mL of aliquot and then vortexed. 5 mL of conc. H_2_SO_4_ was added to the reaction mixture which was later cooled at room temperature. Optical density was read at 485 nm.

### 2.10 Proline estimation

Proline estimation was performed by Bates (1973) method in which fresh leaf material (0.5 g) was taken and homogenized with 10 mL of sulfosalicylic acid with the use of a mortar and pestle, the homogenate was then centrifuged at 10,000 rpm for 10 min. 2 mL of supernatant was taken, and 2 mL each of acid ninhydrin and glacial acetic acid was added, and the mixture was boiled at 100 °C in water bath for 1 h. The test tubes were then placed in an ice bath for 10 min to stop the reaction. To each reaction mixture, 4 mL of toluene was added. The test tubes were vortexed to facilitate quick movement. The toluene layer was separated from the aqueous layer and absorbance was read at 620 nm using UV–Vis spectrophotometer.

### 2.11 Determination of essential mineral elements (Fe, Cu, Mn, Zn, C, P, and S)

A 0.5 g aliquot of oven-dried and finely ground waxy maize kernels was accurately weighed and digested with HNO_3_-H_2_O_2_ in a microwave accelerated reaction system (CEM, Matthews, NC, USA). The concentrations of Zn, Fe, Mn, Ca and Cu in the digested solutions were determined by inductively coupled plasma atomic emission spectroscopy (ICP-AES, OPTIMA 3300 DV, Perkin-Elmer, USA).

### 2.12 Chromium analysis

The Cr content in the seedlings was quantified using the flame atomic absorption spectroscopy method. Fresh soybean seedlings were dried and digested for analysis.

### 2.13 Statistical analysis

The data obtained as mean ± SD of five replicates (n = 5) were subjected to one-way or two-way analysis of variance (ANOVA) followed by *post hoc* analysis using SPSS software (Version 22). The significant difference among mean values was compared using the Duncan’s multiple range test (DMRT) at p≤ 0.05, and lettering was done on all figures.

## 3 Results

### 3.1 Characterization of multi walled CNTs

**Figure 1:**
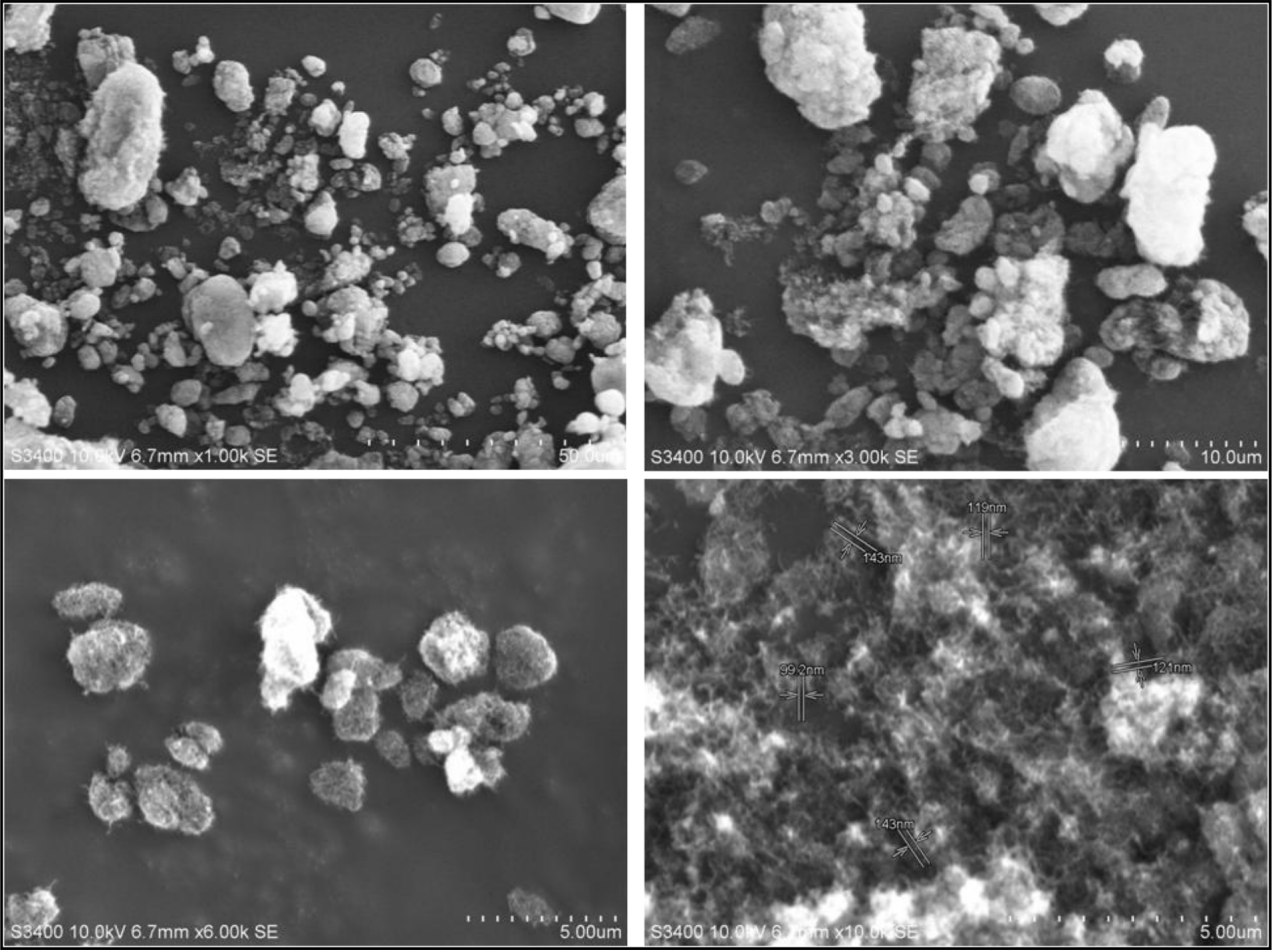
Scanning electron microscopy images of CNTs at different magnifications.

**Figure 1** presents the scanning electron microscopy (SEM) images of MWCNTs captured at different magnifications to observe their surface morphology and structural characteristics. The micrographs reveal a typical entangled and tubular network structure, confirming the formation of CNTs with high aspect ratios. The nanotubes appear as elongated cylindrical structures aggregated into bundles due to strong van der Waals interactions among individual tubes. The SEM images further demonstrate a relatively uniform distribution with smooth surfaces and minimal structural defects, indicating the high purity and well-developed morphology of the MWCNTs.

**Figure 2:**
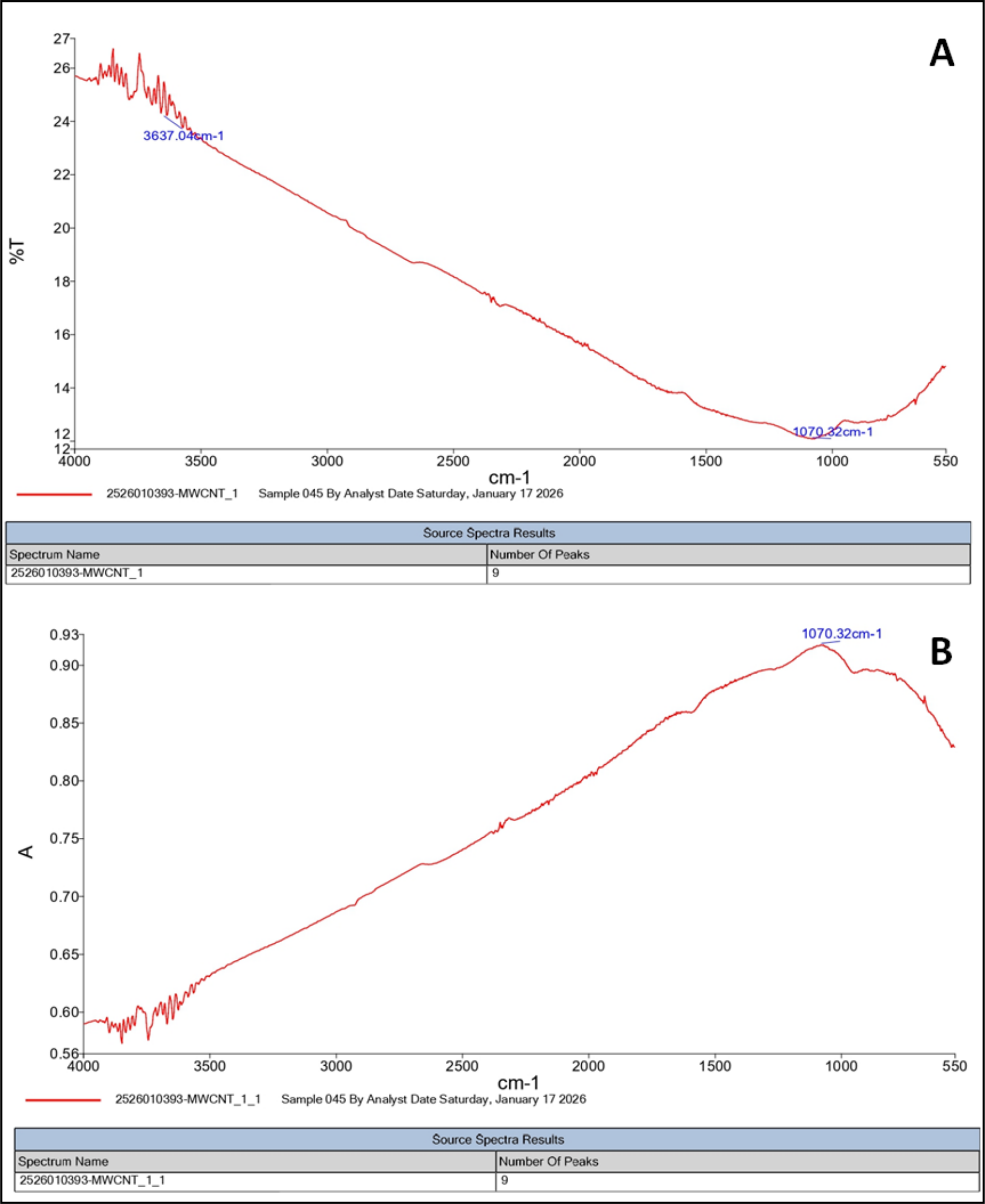
Fourier transform infrared (FTIR) spectrum of CNT **(A, B)**.

**Figure 2** illustrates the Fourier transform infrared (FTIR) spectrum of MWCNTs, which provides information about the functional groups present on the nanotube surface. The spectrum typically shows absorption bands corresponding to various functional groups such as hydroxyl (O–H), carbonyl (C=O), and C–H stretching vibrations. The presence of these peaks indicates the existence of oxygen-containing functional groups on the surface of the carbon nanotubes, which may arise from surface oxidation during synthesis or purification processes. These functional groups enhance the chemical reactivity and dispersion ability of MWCNTs in different media.

**Figure 3:**
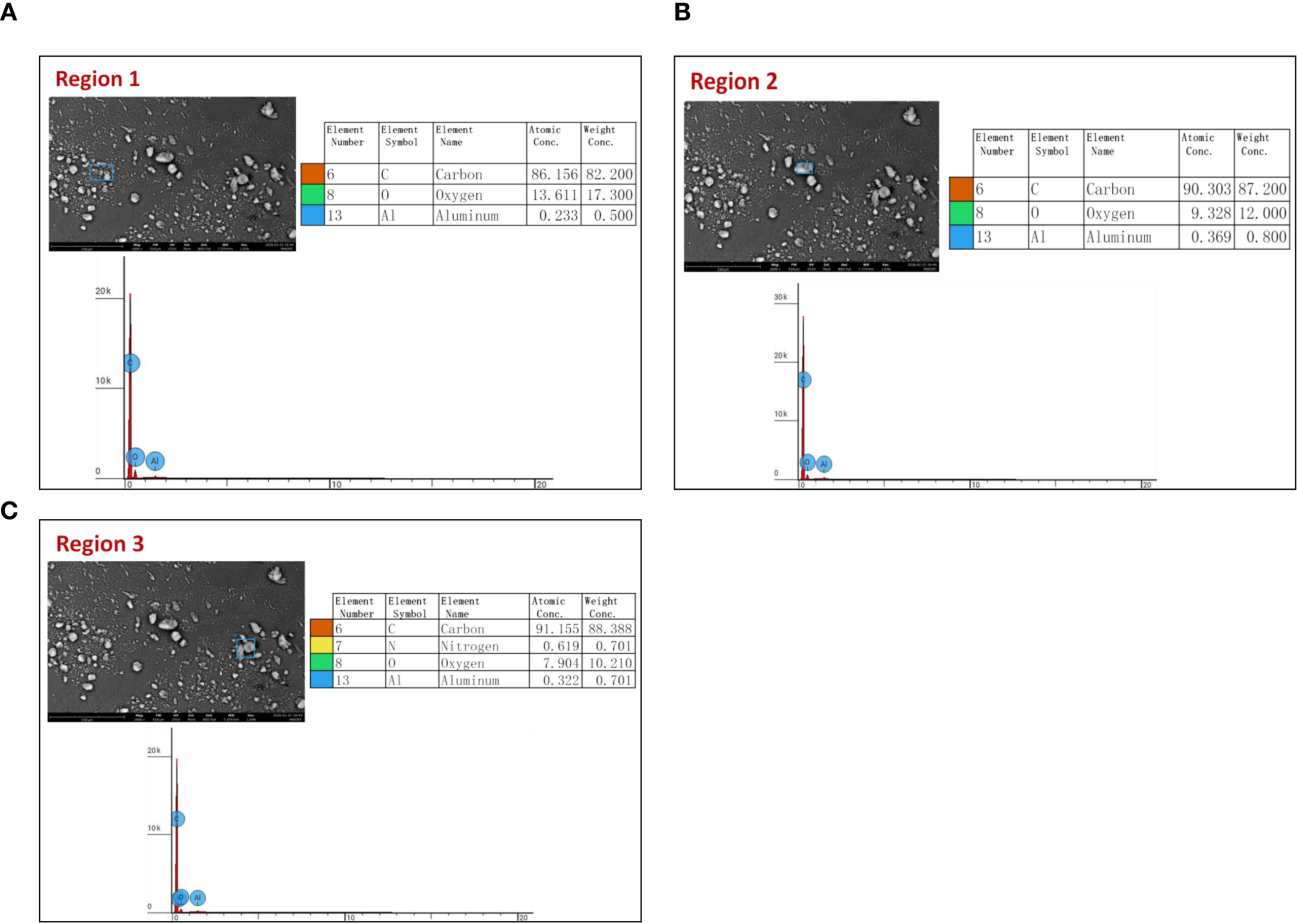
**(A)** Representative SEM image of CNTs indicating region 1 used for energy-dispersive x-ray spectroscopy (EDS) analysis, accompanied by the corresponding elemental spectrum. **(B)** Representative SEM image of CNTs indicating region 2 used for energy-dispersive x-ray spectroscopy (EDS) analysis, accompanied by the corresponding elemental spectrum. **(C)** Representative SEM image of CNTs indicating Region 3 used for energy-dispersive X-ray spectroscopy (EDS) analysis, accompanied by the corresponding elemental spectrum.

**Figures 3A–C** present representative SEM images of carbon nanotubes highlighting three different regions selected for energy-dispersive X-ray spectroscopy (EDS) analysis, along with their corresponding elemental spectra. In **Figure 3A**, Region 1 was analyzed to determine the elemental composition of the nanotube surface, and the EDS spectrum confirms the dominant presence of carbon as the primary element. Similarly, **Figure 3B** shows Region 2 selected for EDS analysis, which also reveals carbon as the major component with minor signals that may correspond to oxygen or trace elements originating from surface functionalization or residual impurities. **Figure 3C** represents Region 3 used for EDS analysis, further confirming the elemental composition and uniform distribution of carbon throughout the nanotube structure. The consistency of elemental signals across the three regions indicates the compositional uniformity and high purity of the CNT sample.

### 3.2 Modulatory effects of CNTs and JA on growth performance of soybean exposed to Cr stress

**Figure 4:**
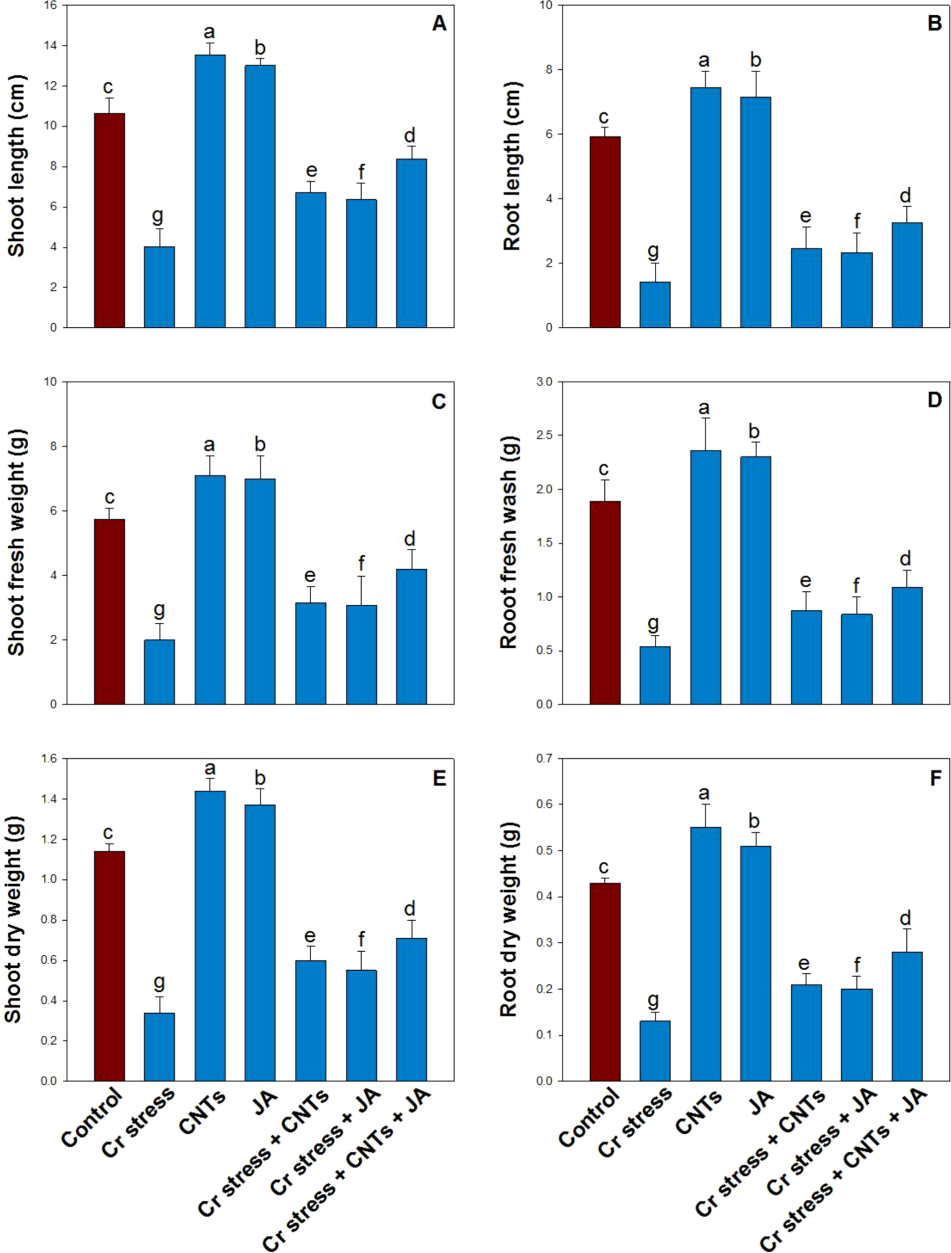
Effect of CNTs and JA on soybean under Cr stress. **(A)** Shoot length, **(B)** Root length, **(C)** Shoot fresh weight, **(D)** Root fresh weight, **(E)** Shoot dry weight, and **(F)** Root dry weight. The data is shown as the average of five sets ± Standard Error (S.E.). The graphs’ bars display distinct characters that indicate a significant difference between the means of the various treatments at P ≤ 0.05 (Tukey’s test).

Chromium stress significantly decreased soybean growth attributes by 68% in shoot length (SL), 76% in root length (RL), 65% in shoot fresh weight (SFW), 71% in root fresh weight (RFW), 70% in shoot dry weight (SDW), and 69% in root dry weight (RDW) compared with the control plants (**Figure 4**). Application of CNTs and JA reversed the toxicity induced by Cr and maintained plant growth attributes. CNTs showed more pronounced effects than JA, increasing SL by 67%, RL by 73%, SFW by 58%, RFW by 62%, SDW by 64%, and RDW by 69% compared with Cr-treated plants. Moreover, the maximum improvement under Cr stress was observed in plants treated with the combined application of CNTs and JA relative to Cr-treated plants.

### 3.3 Effects of CNTs and JA on photosynthetic pigments and their precursors in soybean under Cr stress

**Figure 5:**
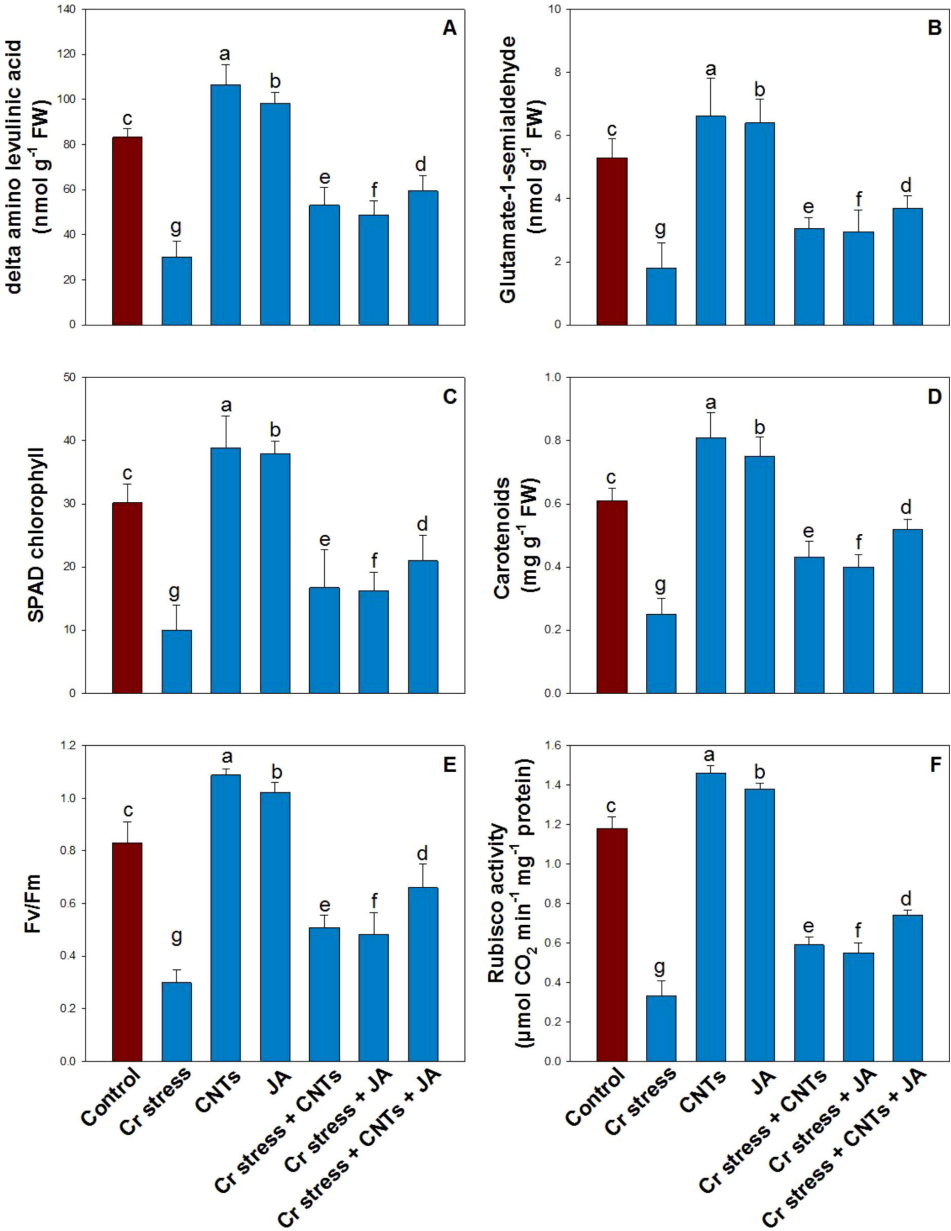
Effect of CNTs and JA on soybean under Cr stress. **(A)** δ-Amino Levulinic acid, **(B)** Glutamate 1-Semialdehyde, **(C)** SPAD chlorophyll, **(D)** Carotenoids, **(E)** Fv/Fm, and **(F)** Rubisco activity. The data is shown as the average of five sets ± Standard Error (S.E.). The graphs’ bars display distinct characters that indicate a significant difference between the means of the various treatments at P ≤ 0.05 (Tukey’s test).

Chromium stress markedly suppressed photosynthetic pigment precursors, pigment content, and photosynthetic efficiency, leading to reductions of 64% in δ-ALA, 66% in GSA, 67% in SPAD chlorophyll values, 58% in carotenoids, 64% in the Fv/Fm, and 72% in RuBisCO activity compared with the control plants (**Figure 5**). In contrast, exogenous application of CNTs and JA effectively alleviated Cr-induced toxicity and restored these physiological and biochemical attributes. Among the individual treatments, CNTs exerted a more pronounced ameliorative effect than JA, resulting in greater improvements across all measured parameters: 28% in δ-ALA, 25% in GSA, 29% in SPAD chlorophyll, 33% in carotenoids, 31% in Fv/Fm, and 24% in RuBisCO activity relative to control plants. Notably, the combined application of CNTs and JA exhibited the strongest mitigation under Cr stress and significantly enhanced all the above parameters.

### 3.4 CNTs and JA-mediated enhancement of photosynthetic performance of soybean under Cr stress

**Figure 6:**
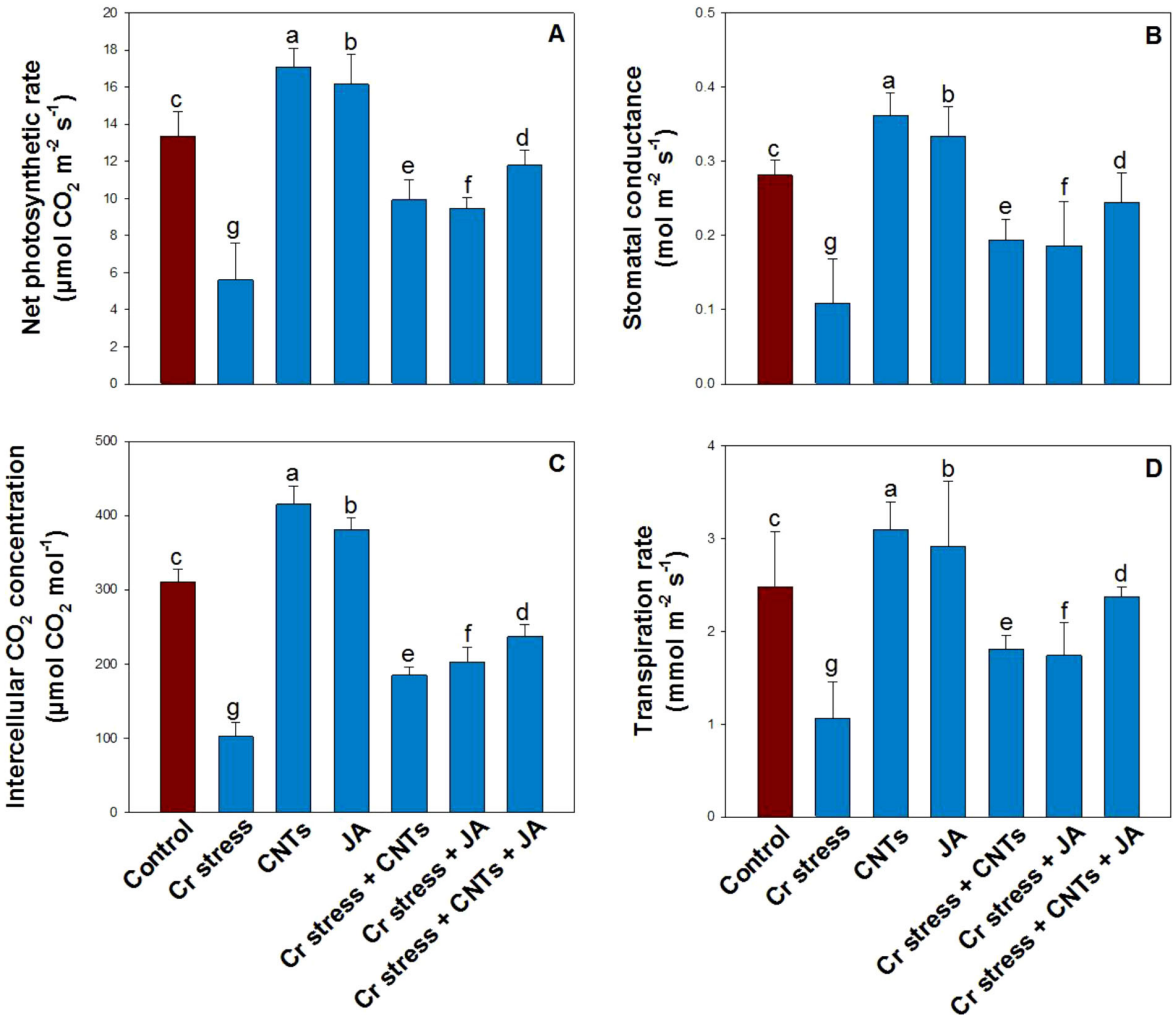
Effect of CNTs and JA on soybean under Cr stress. **(A)** Net photosynthetic rate, **(B)** Stomatal conductance, **(C)** Intercellular CO_2_ concentration, and **(D)** Transpiration rate. The data is shown as the average of five sets ± Standard Error (S.E.). The graphs’ bars display distinct characters that indicate a significant difference between the means of the various treatments at P ≤ 0.05 (Tukey’s test).

Chromium stress significantly reduced P_N_ by 58%, gs by 61%, Ci by 67%, and E by 57% compared with the control plants (**Figure 6**). In contrast, exogenous application of CNTs and JA applied individually or in combination, markedly improved these gas-exchange parameters under both non-stress and Cr-stress conditions. Exogenous application of CNTs and JA increased all the above attributes with P_N_ increasing by 28% and 21%, gs by 29% and 19%, Ci by 34% and 23%, and E by 25% and 18%, relative to control plants.

### 3.5 Effects of CNTs and JA on oxidative stress markers under Cr stress

**Figure 7:**
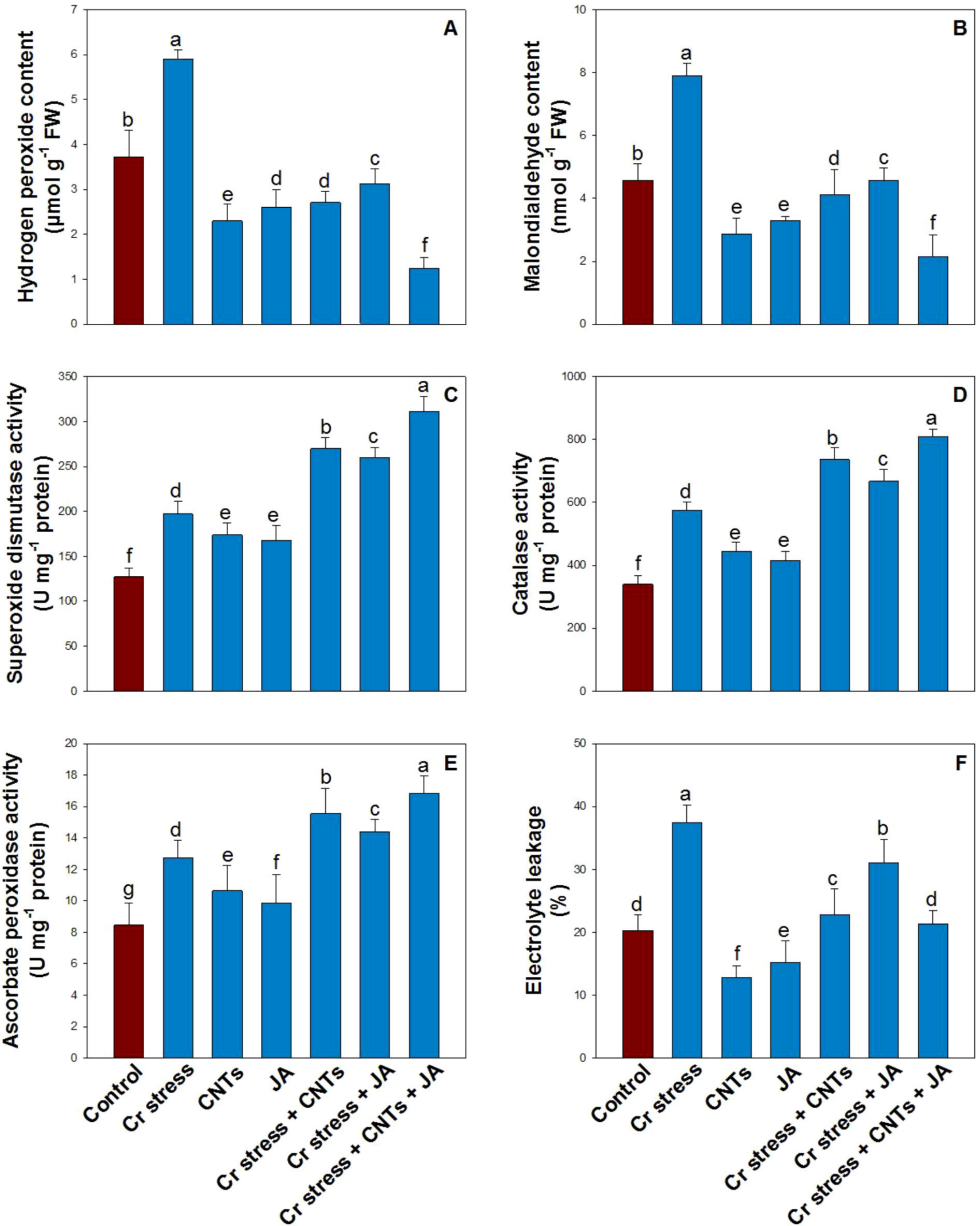
Effect of CNTs and JA on soybean under Cr stress. **(A)** Hydrogen peroxidase, **(B)** Malondialdehyde, **(C)** Superoxide dismutase, **(D)** Catalase, **(E)** Ascorbate peroxidase, and **(F)** Electrolyte leakage. The data is shown as the average of five sets ± Standard Error (S.E.). The graphs’ bars display distinct characters that indicate a significant difference between the means of the various treatments at P ≤ 0.05 (Tukey’s test).

Oxidative stress markers, namely H_2_O_2_ and MDA, were significantly elevated under Cr stress, increasing by 87% and 96%, respectively, compared with the control plants (**Figures 7A, B**). In contrast, exogenous application of CNTs and JA, applied individually or in combination, effectively mitigated Cr-induced oxidative stress, leading to notable reductions in both H_2_O_2_ and MDA levels. The greatest decrease was observed with the combined CNTs and JA treatment, which reduced H_2_O_2_ and MDA contents by 82% and 76%, respectively, relative to Cr-treated plants.

### 3.6 Effects of CNTs and JA on antioxidant enzymes under Cr stress

Chromium stress markedly increased the activities of key antioxidant enzymes, with SOD, CAT, and APX showing increases of 55%, 69%, and 51%, respectively, compared with the control plants (**Figures 7C–E**). Moreover, the exogenous application of CNTs and JA further enhanced the activities of these enzymes. The highest enzymatic activities were recorded in plants subjected to the combined CNTs and JA treatment under Cr stress.

### 3.7 Effects of CNTs and JA on electrolyte leakage under Cr stress

Under Cr stress, EL increased significantly by 84% compared with the control plants (**Figure 7F**), indicating enhanced membrane damage. In contrast, exogenous application of CNTs and JA, applied individually or in combination, effectively reduced EL under Cr stress. EL decreased by 22% with CNTs treatment, 17% with JA application, and 43% under the combined CNTs + JA treatment compared with Cr-stressed plants, indicating a substantial improvement in membrane stability.

### 3.8 Effects of CNTs and JA on cellular metabolites under Cr stress

**Figure 8:**
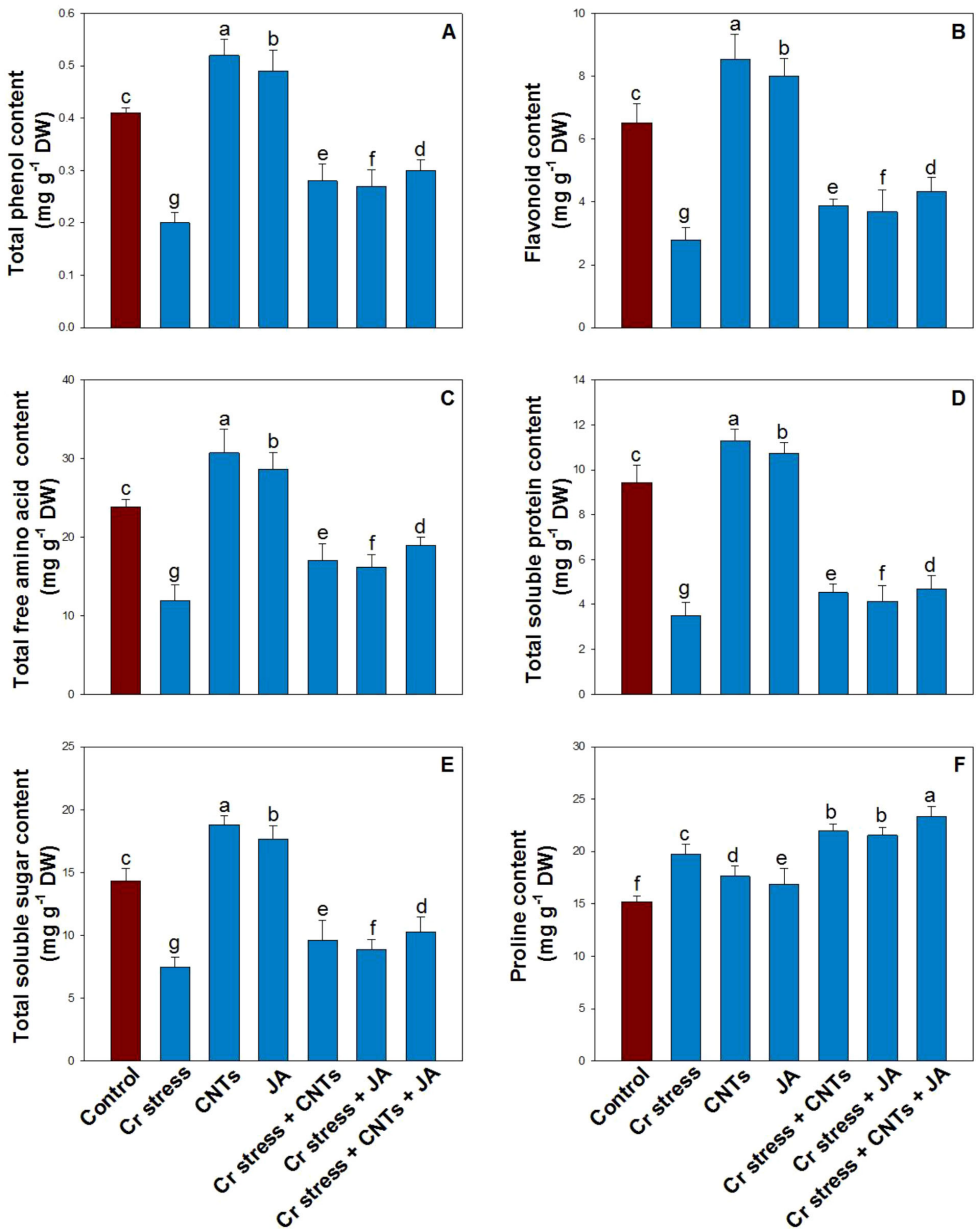
Effect of CNTs and JA on soybean under Cr stress. **(A)** Total phenol, **(B)** Flavonoid, **(C)** Total free amino acid, **(D)** Total soluble protein, **(E)** Total soluble sugar, and **(F)** Proline. The data is shown as the average of five sets ± Standard Error (S.E.). The graphs’ bars display distinct characters that indicate a significant difference between the means of the various treatments at P ≤ 0.05 (Tukey’s test).

Chromium stress significantly decreased the accumulation of key metabolites, including total phenols by 51%, flavonoids by 57%, free amino acids by 50%, total soluble sugars by 48%, and total soluble proteins by 63%, compared with the control plants (**Figures 8A–E**). In contrast, exogenous application of CNTs and JA, applied individually or in combination, effectively reversed these Cr-induced metabolic impairments. The greatest enhancement under Cr stress was observed in plants treated with the combined application of CNTs and JA, showing markedly higher metabolite levels relative to plants exposed to Cr alone.

### 3.9 Effects of CNTs and JA on proline content under Cr stress

Proline content increased significantly under Cr stress, showing a 30% elevation relative to the control plants (**Figure 8F**). Exogenous application of CNTs and JA under non-stress conditions resulted in modest increases in proline accumulation, by 16% and 11%, respectively, compared with the control. Notably, the combined application of CNTs and JA under Cr stress induced the highest proline accumulation, enhancing proline content by 18% relative to Cr-treated plants, suggesting an improved osmoprotective response.

### 3.10 Effect of CNTs and JA on essential mineral elements (Fe, Cu, Mn, Zn, C, P, and S)

**Figure 9:**
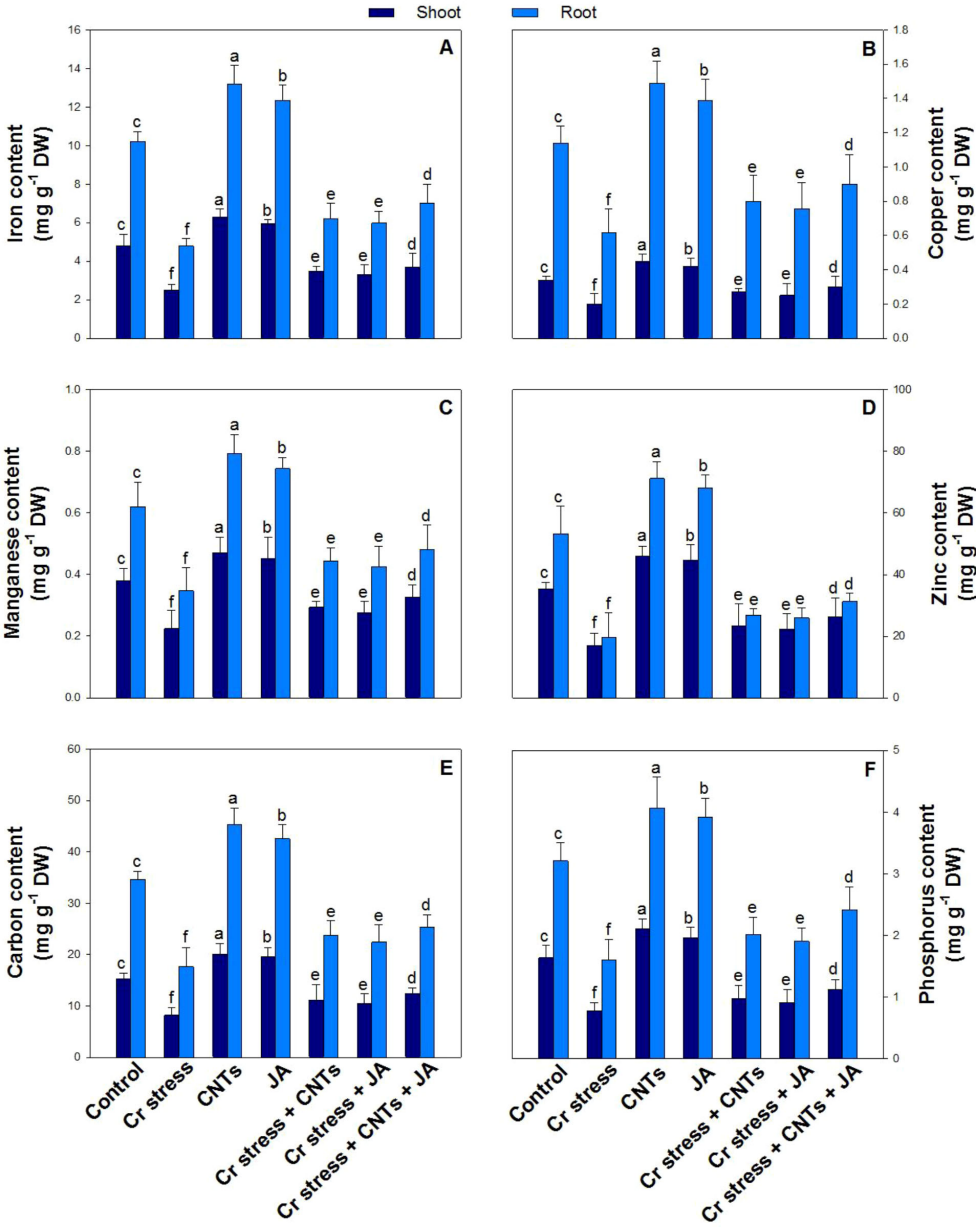
Effect of CNTs and JA on soybean under Cr stress. **(A)** Iron, **(B)** Copper, **(C)** Manganese, **(D)** Zinc, **(E)** Carbon, and **(F)** Phosphorus. The data is shown as the average of five sets ± Standard Error (S.E.). The graphs’ bars display distinct characters that indicate a significant difference between the means of the various treatments at P ≤ 0.05 (Tukey’s test).

**Figure 10:**
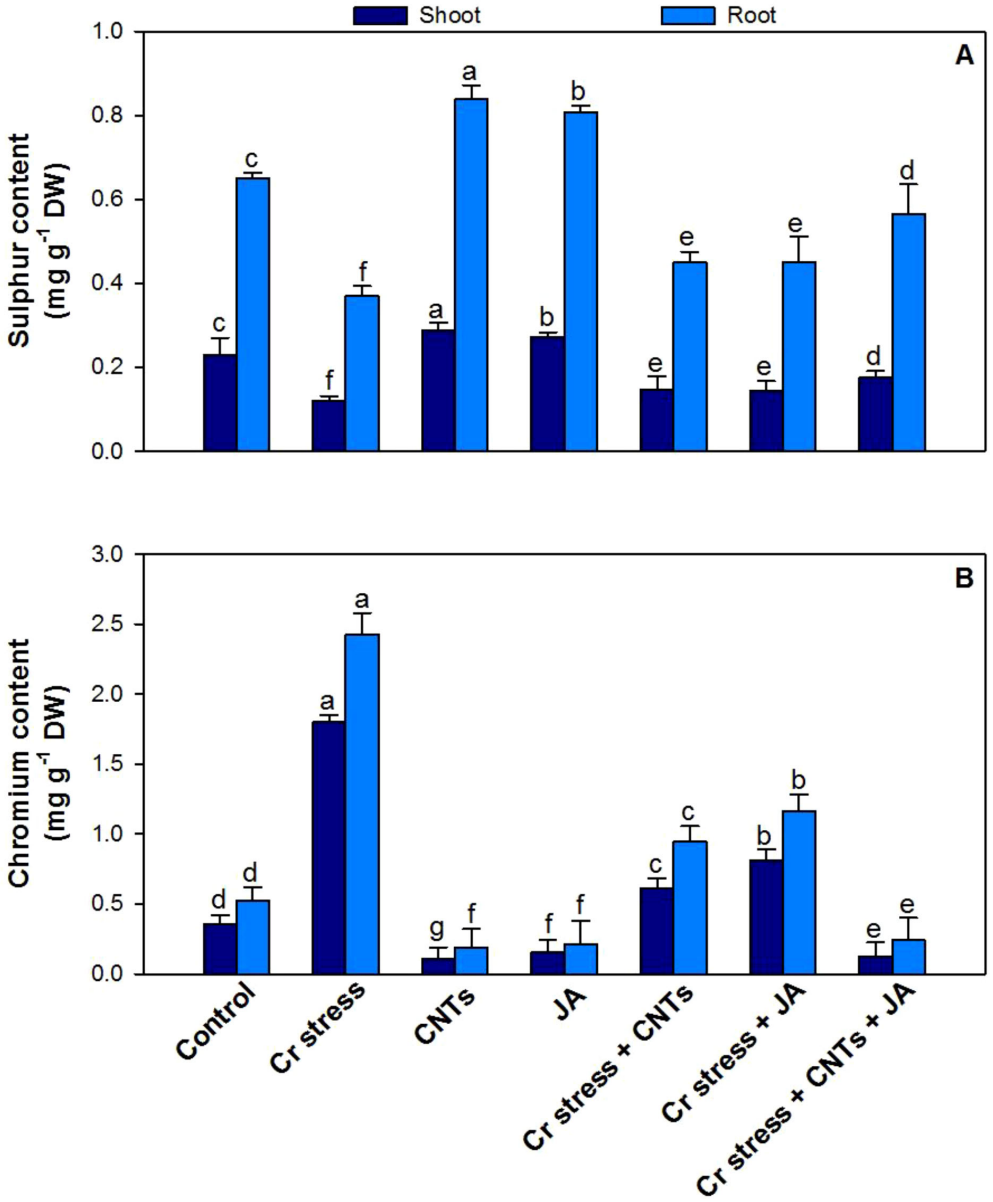
Effect of CNTs and JA on soybean under Cr stress. **(A)** Sulphur and **(B)** Chromium. The data is shown as the average of five sets ± Standard Error (S.E.). The graphs’ bars display distinct characters that indicate a significant difference between the means of the various treatments at P ≤ 0.05 (Tukey’s test).

Chromium stress significantly disrupted the accumulation of essential mineral elements in both shoots and roots, resulting in marked reductions of Fe by 48% and 53%, Cu by 39% and 46%, Mn by 41% and 44%, Zn by 52% and 63%, C by 46% and 50%, P by 53% and 49%, and S by 47% and 42%, respectively, compared with the control plants (**Figures 9**, **10A**). In contrast, exogenous application of CNTs and JA, applied individually or in combination, effectively alleviated Cr-induced nutrient imbalances and restored mineral concentrations. The maximum ameliorative effect under Cr stress was observed with the combined application of CNTs and JA, which significantly increased Fe by 46% and 47%, Cu by 53% and 48%, Mn by 46% and 39%, Zn by 55% and 59%, C by 51% and 44%, P by 46% and 51%, and S by 45% and 53% in shoots and roots, respectively, relative to Cr-treated plants.

### 3.11 Effect of CNTs and JA on Cr toxicity

Exogenous application of CNTs and JA significantly reduced Cr accumulation, resulting in decreases of 61% and 52%, respectively, compared with Cr-treated plants (**Figure 10B**). The maximum reduction in Cr content was observed in plants receiving the combined application of CNTs and JA, which lowered Cr accumulation by 90% relative to plants exposed to Cr alone.

## 4 Discussion

Researchers are becoming increasingly alarmed by the rising Cr contamination in air, soil, and water. Exposure to Cr induces multiple toxicity symptoms in plants, including inhibited growth, leaf chlorosis, and a decline in overall biomass. Soybean, a major agricultural crop, is especially vulnerable to Cr stress (Chen et al., 2023). Therefore, supplementation of CNTs and JA on soybean plants under Cr exposure could improve our understanding of stress responses and support the development of Cr-tolerant cultivars an area that remains largely underexplored.

Chromium contamination is a major abiotic stress that negatively influences plant growth and development. Cr toxicity disrupts cellular structure and function, leading to restricted cell division and elongation. As a result, common growth indices such as shoot length, root length, fresh and dry biomass are significantly reduced under Cr exposure (Shiji et al., 2025). Cr accumulation in roots inhibits root cell elongation by altering cell wall integrity and inducing oxidative stress, which limits water and nutrient uptake and ultimately stunts overall plant growth. Application of CNTs has emerged as a promising approach to mitigating heavy metal stress in plants due to their unique physicochemical properties and ability to interact at the molecular level with plant tissues (Wani et al., 2024; Safdar et al., 2022). CNTs can ameliorate metal toxicity through multiple mechanisms, including improved nutrient acquisition, enhanced antioxidant enzyme activity, and adsorption or immobilization of toxic metal ions. In our study, treatment with CNTs under Cr stress significantly improved shoot and root length, as well as plant fresh and dry weight relative to Cr alone (**Figure 4**). These results corroborate previous findings where CNTs enhanced plant growth under metal stress (Chen et al., 2021). For instance, Hao et al. (2023) reported that CNTs improved root elongation and biomass accumulation in maize by facilitating water uptake and stimulating cell division. Jasmonic acid is a well-documented plant growth regulator that mediates responses to various abiotic stresses, including heavy metals. JA participates in stress signaling pathways that activate defense-related gene expression, enhance antioxidant defense, and regulate ion homeostasis. Under Cr stress, JA application can mitigate adverse effects by reinforcing stress tolerance and maintaining growth homeostasis (Ali et al., 2023). The synergistic alleviation of Cr toxicity by CNTs and JA suggests a coordinated enhancement of physiological and biochemical processes. CNTs can facilitate better JA transport within plant tissues, optimizing JA’s hormonal effectiveness under stress. Moreover, the combined treatment may further stabilize membrane integrity and reduce lipid peroxidation by potentiating antioxidant enzyme activities, leading to improved cellular growth and biomass accumulation (Kumar et al., 2024).

Chromium stress markedly impaired photosynthetic metabolism by disrupting both pigment biosynthesis and photochemical efficiency, which was evident from the significant reductions in δ-aminolevulinic acid (δ-ALA), glutamate-1-semialdehyde (GSA), SPAD chlorophyll values, total chlorophyll, carotenoids, Fv/Fm, and Rubisco activity (Kaya et al., 2023). The decline in δ-ALA and GSA suggests that Cr strongly inhibited early steps of the tetrapyrrole pathway, ultimately limiting chlorophyll synthesis and causing chlorosis, while the reduction in carotenoids further weakened photoprotection and increased vulnerability to oxidative damage. Decreased Fv/Fm under Cr exposure indicates damage to PSII reaction centers and impaired electron transport, which likely enhanced ROS formation and aggravated pigment degradation (Tan et al., 2025; Chakhmakhcyan et al., 2026). In parallel, Rubisco activity was substantially reduced, reflecting Cr-induced inhibition of Calvin cycle enzymes, reduced CO_2_ fixation efficiency, and oxidative modification of photosynthetic proteins (Liu et al., 2024). These results are consistent with earlier reports showing that Cr toxicity induces oxidative stress, damages chloroplast ultrastructure, and suppresses pigment formation and carbon assimilation, leading to pronounced photosynthetic inhibition (Gill and Tuteja, 2010). The application of CNTs and JA, particularly in combination, effectively alleviated Cr-mediated photosynthetic damage by restoring chlorophyll precursor metabolism and maintaining photosystem stability. CNTs significantly improved δ-ALA, GSA, SPAD chlorophyll, total chlorophyll, carotenoids, Fv/Fm, and Rubisco activity, likely due to improved nutrient uptake, stabilization of chloroplast membranes, reduced Cr bioavailability, and enhanced physiological performance under stress. Likewise, JA application enhanced these traits by activating stress-responsive signaling and antioxidant defenses, reducing ROS-induced pigment degradation, and supporting the integrity of PSII and Calvin cycle processes (Julin et al., 2025). Notably, the combined CNTs + JA treatment produced the greatest recovery in pigments, Fv/Fm, and Rubisco activity, indicating a synergistic interaction where CNTs primarily improved ionic and physiological balance while JA reinforced cellular defense regulation (Abd Elgawad et al., 2020). This integrated protection of pigment biosynthesis and carbon fixation machinery explains the improved photosynthetic competence and stress tolerance of plants under Cr toxicity (Ghorbani et al., 2024).

Chromium stress caused a pronounced decline in gas-exchange traits, including P_N_, gs, E, and Ci, reflecting severe limitations in both stomatal regulation and metabolic carbon assimilation (Fusaro et al., 2025). The reduction in gs and E under Cr stress indicates partial stomatal closure, which restricts CO_2_ diffusion into leaves and disrupts leaf water relations (Kumar et al., 2022). This stomatal limitation, coupled with Cr-induced damage to chloroplast membranes and PSII activity, ultimately suppresses Pn. In many cases, altered Ci under Cr stress is associated with combined stomatal and non-stomatal constraints: stomatal closure initially lowers Ci, whereas prolonged inhibition of Rubisco activity and electron transport can lead to abnormal Ci patterns due to reduced CO_2_ fixation efficiency (Morales et al., 2020). Application of CNTs and JA, especially their combined treatment, significantly improved P_N_, gs, Ci, and E compared to Cr-stressed plants. CNTs likely contributed by improving root water uptake, nutrient acquisition, and maintaining membrane stability, which supports stomatal functioning and enhances CO_2_ diffusion and photosynthetic performance under metal stress (Martínez-Ballesta et al., 2016). Meanwhile, JA improved gas-exchange efficiency by strengthening antioxidant defenses, reducing oxidative damage in guard cells and chloroplasts, and sustaining Calvin cycle activity, thereby restoring Pn and optimizing Ci regulation. The strongest recovery in Pn with relatively improved gs and E under CNTs + JA suggests that the combined strategy alleviated both stomatal and biochemical limitations, maintaining CO_2_ assimilation and water-use processes under Cr toxicity (Agnihotri and Seth, 2020; Alp et al., 2023).

**Figure 11:**
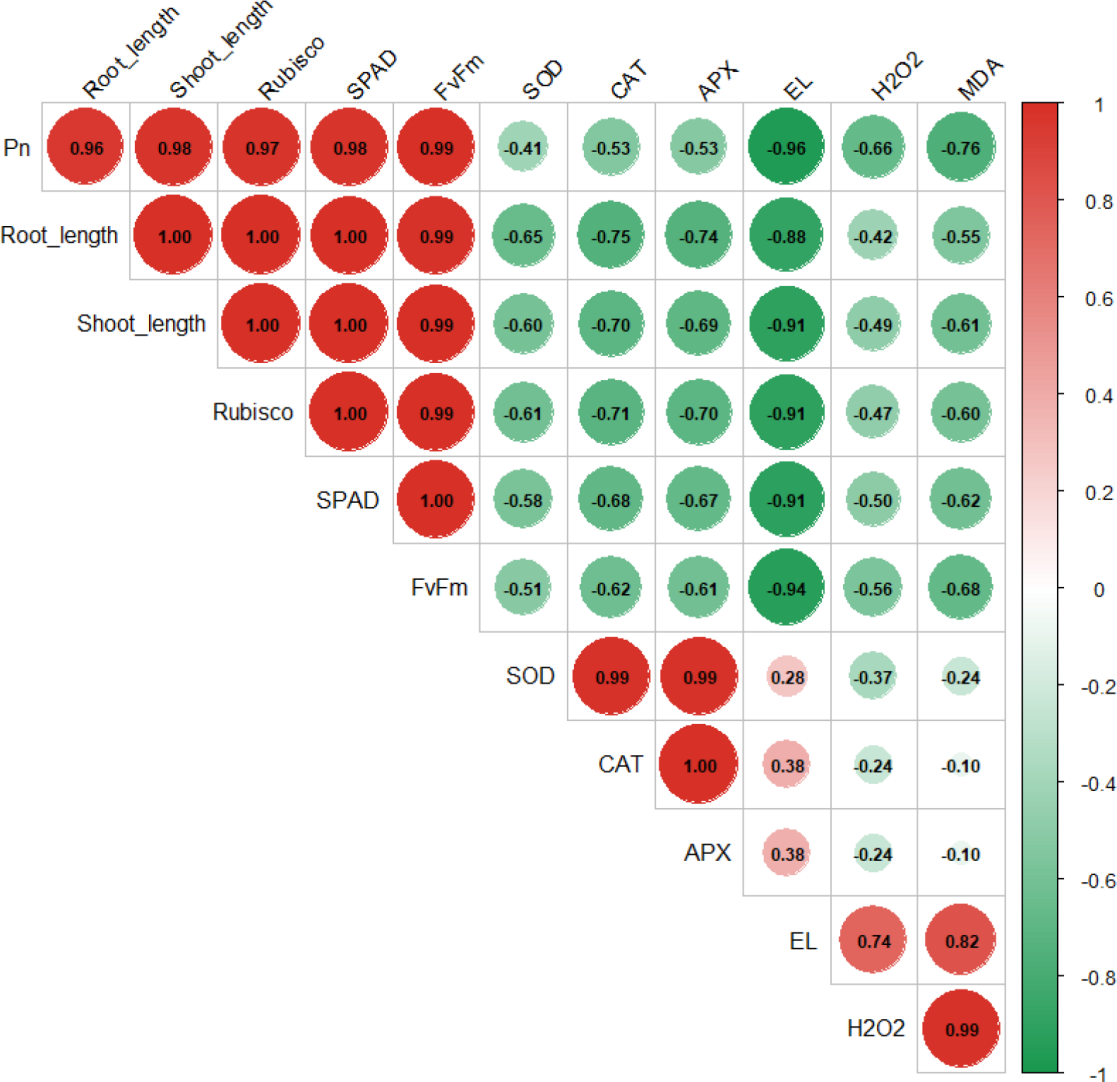
Pearson correlation matrix illustrating relationships among growth, photosynthetic, antioxidant, and oxidative stress parameters across all treatments. Circle size and color intensity represent the strength and direction of Pearson correlation coefficients (r), with red indicating positive correlations and green indicating negative correlations.

The present results indicate pronounced oxidative injury in Cr-exposed soybean, as shown by elevated H_2_O_2_ and MDA accumulation along with increased EL. Malondialdehyde is widely recognized as a reliable indicator of membrane lipid peroxidation (Parvin et al., 2021). Although Cr is a non-essential and highly toxic metal, it does not directly trigger oxidative stress through the Haber–Weiss reaction. Instead, Cr disrupts cellular redox balance by weakening the antioxidant defense machinery and stimulating excessive ROS formation, which ultimately accelerates membrane lipid peroxidation (Ali et al., 2015; Mahmud et al., 2018). The overproduction of ROS enhances MDA formation and promotes oxidative deterioration of key biomolecules, including proteins, lipids, and nucleic acids. The increase in EL further reflects membrane destabilization and injury, which is closely linked with enhanced lipid peroxidation. Similar oxidative damage under Cr stress has also been reported in maize (Malik et al., 2022). In contrast, the combined application of CNTs and JA markedly alleviated Cr-induced oxidative stress, as evidenced by reduced H_2_O_2_ and MDA contents and lower EL in soybean. Antioxidant enzymes such as SOD, CAT, and APX play a central role in ROS detoxification (Qureshi et al., 2020). Superoxide dismutase acts as the first line of defense by converting superoxide radicals (O_2_^.–^) into H_2_O_2_, which is subsequently detoxified into water by CAT and APX (Ashraf et al., 2022). Therefore, the CNTs- and JA-induced enhancement of antioxidant enzyme activity appears to be a key mechanism contributing to improved chromium tolerance in soybean (**Figure 11**). These observations align with earlier findings where improved antioxidant defense capacity significantly reduced Cr toxicity in castor bean (Qureshi et al., 2020).

Chromium stress significantly altered the non-enzymatic antioxidant system and osmolyte metabolism, as reflected by notable changes in total phenolics, flavonoids, free amino acids, total soluble protein, total soluble sugars, and proline (Ali et al., 2025). Under Cr toxicity, plants typically enhance phenolic and flavonoid accumulation as a protective strategy because these compounds act as strong ROS scavengers and metal chelators, helping to limit oxidative injury and stabilize cellular structures (Qureshi et al., 2024). Similarly, proline and soluble sugars increase under Cr stress to maintain osmotic balance, protect membranes, and support redox buffering, while elevated FAA levels indicate stress-induced protein turnover and metabolic adjustment (Vettore et al., 2021). In contrast, TSP often declines under heavy metal stress due to inhibited protein synthesis, enhanced protease activity, and oxidative modification of proteins, reflecting overall metabolic impairment and growth restriction under Cr exposure (Shahid et al., 2026; Hassan and Su, 2026). The application of CNTs and JA, particularly in combination, further strengthened these biochemical defense responses and improved metabolic stability under Cr stress. CNTs enhanced the accumulation of phenolics and flavonoids and promoted higher TSS and proline levels, likely by improving nutrient uptake, reducing Cr-induced damage, and supporting carbon metabolism (Shi et al., 2023; Hao et al., 2023). Meanwhile, JA is well recognized for inducing secondary metabolites and stress-responsive osmolytes, leading to greater accumulation of phenolics, flavonoids, and proline, while also helping restore TSP by protecting cellular machinery and reducing oxidative damage (Wu et al., 2024). The combined CNTs + JA treatment generally resulted in the most balanced biochemical profile, characterized by higher antioxidant metabolites and osmoprotectants with improved protein stability, indicating synergistic regulation of both ROS detoxification and osmotic adjustment pathways. This integrated metabolic strengthening likely contributed to improved growth and photosynthetic performance of plants under Cr toxicity (Ikram et al., 2025).

Chromium stress significantly disturbed mineral nutrition, resulting in marked reductions in essential macro- and micronutrients such as Fe, Cu, Mn, Zn, and P in plant tissues (Bhat et al., 2023; Nikolaou et al., 2022). This decline is mainly attributed to Cr competition with nutrient uptake sites, damage to root membranes, inhibition of transporter activities, and reduced root growth, which collectively restrict the absorption and translocation of nutrients (Zulfiqar et al., 2023). Among these, Fe and Mn are especially sensitive under Cr toxicity because they are directly linked with chlorophyll biosynthesis and photosynthetic electron transport; thus, their depletion contributes to chlorosis, reduced SPAD values, and impaired photosynthetic efficiency (Smythers et al., 2023). Similarly, Zn and Cu deficiencies under Cr stress weaken enzymatic and antioxidant functions, while reduced P uptake limits ATP synthesis, membrane phospholipid stability, and energy-dependent metabolic processes, ultimately contributing to poor growth and biomass production (Jurowski et al., 2014). The application of CNTs and JA, particularly in combination, substantially improved Fe, Cu, Mn, Zn, and P levels under Cr stress, indicating restoration of nutrient uptake and ionic homeostasis (Gupta and Saharan, 2026; El-Saadony et al., 2022). CNTs likely enhanced nutrient acquisition by improving root functionality, water uptake, and rhizospheric nutrient availability, while also reducing Cr bioavailability and limiting Cr-induced membrane injury (Ghorbani et al., 2024). In addition, CNTs may facilitate nutrient transport through improved root hydraulic conductivity and enhanced physiological activity (Liu et al., 2026). JA further supported nutrient balance by strengthening root metabolism, regulating stress-responsive transport systems, and reducing oxidative damage to root tissues, thereby maintaining nutrient absorption and translocation under stress conditions (Hasanuzzaman et al., 2020; Alam et al., 2025). The combined CNTs + JA treatment produced the greatest improvement in nutrient contents, suggesting a synergistic mitigation mechanism where CNTs improve physical uptake capacity and reduce Cr interference, while JA regulates cellular defense and transporter-level responses.

**Figure 12:**
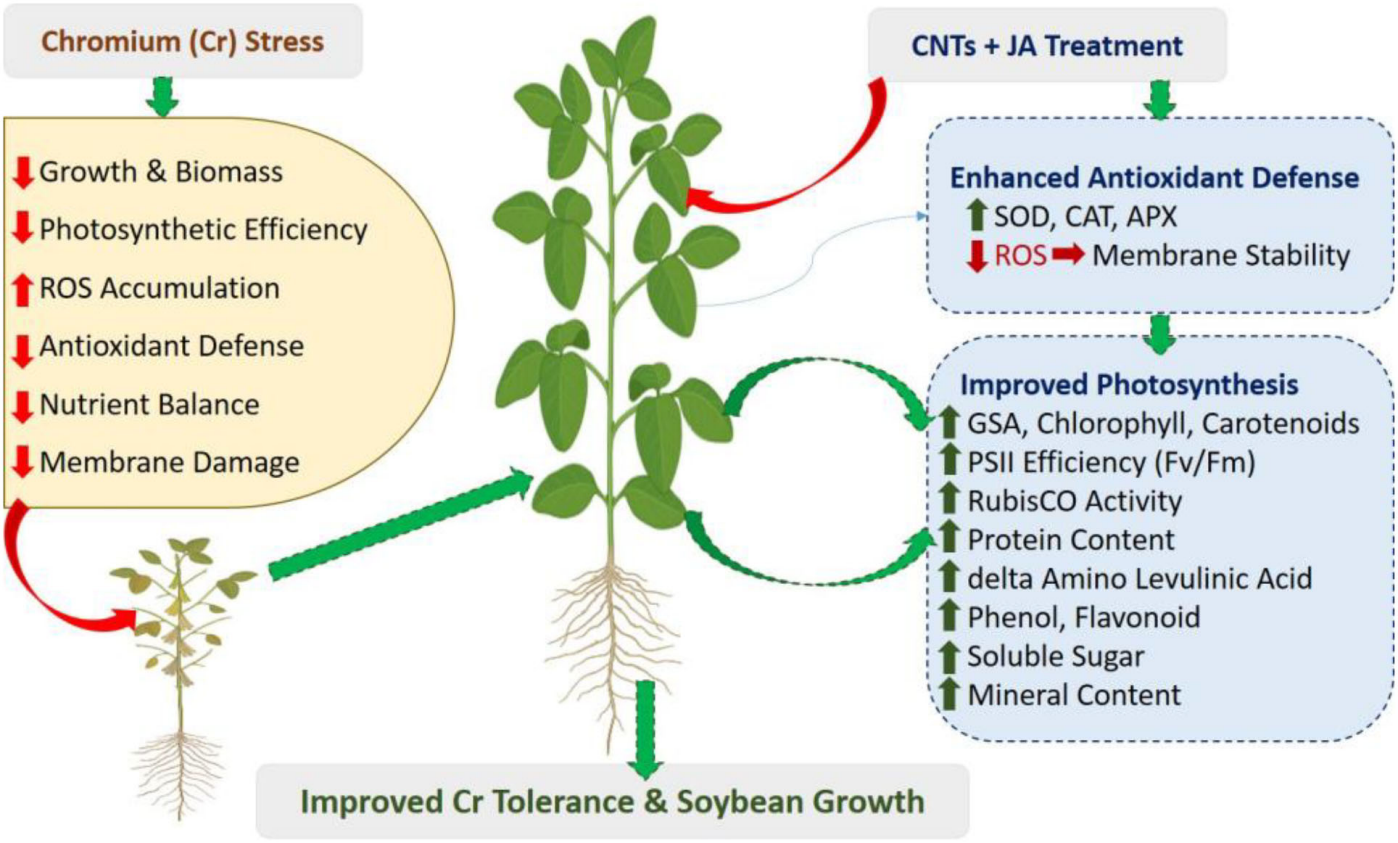
Schematic illustration showing the protective role of CNTs and JA under Cr-induced stress.

Improved nutrient uptake in this study can be explained by the combined physiological and biochemical roles of CNTs and JA in alleviating Cr toxicity and restoring root functionality. Under Cr stress, nutrient absorption is strongly inhibited because Cr damages root membranes, suppresses root growth, and interferes with nutrient transporters responsible for the uptake of essential mineral elements. The application of CNTs likely enhanced nutrient acquisition by improving root architecture, increasing root surface area, and enhancing water permeability, which collectively facilitate better absorption of nutrients from the growth medium. CNTs may also reduce Cr bioavailability through adsorption or immobilization processes in the rhizosphere, thereby decreasing Cr interference with nutrient uptake systems. Chromium also directly interferes with the absorption of Fe and Mn. Cr, particularly in its hexavalent form, competes with Fe and Mn for uptake through shared transport systems present in root cell membranes. Because Fe and Mn are taken up through divalent metal transporters, the presence of excess Cr disrupts these transport pathways and reduces the efficiency of Fe and Mn absorption. Additionally, Cr-induced oxidative stress damages root membrane integrity and transporter proteins, further limiting Fe and Mn uptake. The reduced availability of Fe and Mn directly affects chlorophyll biosynthesis and the photosynthetic electron transport chain, leading to chlorosis, lower SPAD values, and reduced photosynthetic efficiency (**Figure 12**).

Furthermore, CNTs may enhance nutrient translocation by improving root hydraulic conductivity and stimulating physiological activities within plant tissues. At the same time, JA plays an important regulatory role in stress signaling and plant defense responses. JA helps maintain membrane stability, regulates the expression of nutrient transporter proteins, and strengthens antioxidant defense systems, thereby protecting root tissues from oxidative damage caused by Cr stress. This protection allows plants to maintain active nutrient absorption and translocation processes. Therefore, the combined application of CNTs and JA exerts a synergistic effect: CNTs enhance the physical capacity of roots to absorb nutrients and reduce Cr interference, while JA regulates cellular defense mechanisms and transporter activity. Together, these mechanisms restore ionic homeostasis and significantly improve the uptake of essential nutrients such as Fe, Mn, Zn, Cu, and P, ultimately supporting better plant growth and metabolic performance under chromium stress conditions.

## 5 Conclusion

In conclusion, Cr stress markedly reduced soybean growth and photosynthetic efficiency by disturbing redox homeostasis and mineral nutrient balance. However, pre-treatment with CNTs and JA effectively alleviated Cr-induced toxicity. The combined application significantly enhanced antioxidant defense by increasing the activities of SOD, CAT, and APX, thereby limiting oxidative damage and improving membrane stability. Moreover, CNTs and JA preserved photosynthetic performance through stimulation of chlorophyll biosynthesis and pigment stability, reflected in higher levels of GSA, δ-ALA, chlorophyll, carotenoids, and improved PSII efficiency (Fv/Fm). These improvements were further supported by the recovery of gas-exchange parameters (PN and gs) and enhanced Rubisco activity, indicating alleviation of both stomatal and non-stomatal limitations to carbon assimilation. Overall, the synergistic action of CNTs and JA strengthened antioxidant metabolism and maintained photosynthetic machinery, leading to improved Cr tolerance in soybean. This study highlights the potential of nano-enabled hormonal strategies as a promising and sustainable approach for enhancing crop resilience and productivity in Cr-contaminated agroecosystems.

## Data Availability

The original contributions presented in the study are included in the article/supplementary material. Further inquiries can be directed to the corresponding author.
